# Estimating Influenza Outbreaks Using Both Search Engine Query Data and Social Media Data in South Korea

**DOI:** 10.2196/jmir.4955

**Published:** 2016-07-04

**Authors:** Hyekyung Woo, Youngtae Cho, Eunyoung Shim, Jong-Koo Lee, Chang-Gun Lee, Seong Hwan Kim

**Affiliations:** ^1^ Department of Health Science and Service School of Public Health Seoul National University Seoul Republic Of Korea; ^2^ Global Medical Center School of Medicine Seoul National University Seoul Republic Of Korea; ^3^ Real-time Ubiquitous System Laboratory Department of Computer Science and Engineering Seoul National University Seoul Republic Of Korea; ^4^ Search SU Datamining Team Daum Kakao Incorporated Seoul Republic Of Korea

**Keywords:** influenza, surveillance, population surveillance, infodemiology, infoveillance, Internet search, query, social media, big data, forecasting, epidemiology, early response

## Abstract

**Background:**

As suggested as early as in 2006, logs of queries submitted to search engines seeking information could be a source for detection of emerging influenza epidemics if changes in the volume of search queries are monitored (infodemiology). However, selecting queries that are most likely to be associated with influenza epidemics is a particular challenge when it comes to generating better predictions.

**Objective:**

In this study, we describe a methodological extension for detecting influenza outbreaks using search query data; we provide a new approach for query selection through the exploration of contextual information gleaned from social media data. Additionally, we evaluate whether it is possible to use these queries for monitoring and predicting influenza epidemics in South Korea.

**Methods:**

Our study was based on freely available weekly influenza incidence data and query data originating from the search engine on the Korean website Daum between April 3, 2011 and April 5, 2014. To select queries related to influenza epidemics, several approaches were applied: (1) exploring influenza-related words in social media data, (2) identifying the chief concerns related to influenza, and (3) using Web query recommendations. Optimal feature selection by least absolute shrinkage and selection operator (Lasso) and support vector machine for regression (SVR) were used to construct a model predicting influenza epidemics.

**Results:**

In total, 146 queries related to influenza were generated through our initial query selection approach. A considerable proportion of optimal features for final models were derived from queries with reference to the social media data. The SVR model performed well: the prediction values were highly correlated with the recent observed influenza-like illness (*r*=.956; *P*<.001) and virological incidence rate (*r*=.963; *P*<.001).

**Conclusions:**

These results demonstrate the feasibility of using search queries to enhance influenza surveillance in South Korea. In addition, an approach for query selection using social media data seems ideal for supporting influenza surveillance based on search query data.

## Introduction

An early and now well-known example of utilizing Internet data for a health-related applications came from the estimation of influenza incidence using anonymous logs of Web search engine queries. First proposed in 2006 by Eysenbach under the umbrella term “infodemiology”, numerous recent studies have added further evidence of a correlation between search query data from Google [1-3], Yahoo! [4], Baidu [5], or other medical websites [6] and traditional data used for influenza surveillance, such as influenza-like illness (ILI) and/or laboratory-confirmed data. These studies indicate that individuals faced with disease or ill health will search for information on the Internet regarding their state of health and possible countermeasures to illness; logs of queries submitted to search engines by individuals seeking this information are potential sources of information for detecting emerging epidemics, as it is possible to track changes in the volumes of specific search queries. However, the recent errors arising from Google Flu Trends, which has been predominantly used in previous studies, serves as a reminder to investigators that this novel data paradigm calls for critical assessment and the development of more empirical methodologies to explore the predictive utility of big data [7,8]. It is clear that current and future studies need to focus on methods to more precisely identify the particular phases associated with influenza epidemics based on data from these highly informative sources.

Selecting the queries that are most likely to be associated with influenza epidemics poses a particular challenge for the generation of improved predictions. In previous studies, researchers have utilized queries selected by various methods, such as specific keyword tools offered by particular websites [5], surveys of patients who visited the emergency room [1,9], or common knowledge about influenza including the definition of ILI [9,10], as well as fully automated methods for identifying queries related to influenza from search logs [3,4,6]. Because researchers do not have full access to search logs, an approach using social media data may also be helpful for obtaining information for query selection. Recently, social media data have been highlighted as an additional potential data source for disease surveillance because they contain a greater variety of contextual health information with diverse descriptions of health states. Thus, it could be a useful reference point for researchers who wish to select initial target queries in query-based prediction.

In South Korea, there is currently no forecasting system for infectious disease based on search query data [1,9], despite the high availability and use of the Internet in Korea [11]. Moreover, few studies thus far have evaluated whether such data could be of value in national influenza forecasting [1,9], and a recent study has suggested that Google Trends in the Korean language is insufficient for use as a model for influenza prediction in South Korea [1]. We need to proactively determine whether queries of search engines that are more widely used by Koreans have the capacity to enhance traditional influenza surveillance systems in South Korea. We consider the use of social media data to select queries that are most likely to be associated with influenza epidemics in a situation involving limited access to search logs. An attempt to exploit the complementary nature of two types of data sources could result in a rapid and efficient prediction of the occurrence of influenza and their proliferation, thereby allowing for better recognition of influenza and initiation of preventive measures.

The purpose of this study was to further explore two concerns: (1) to describe a methodological extension for detecting influenza outbreaks using search query data, providing a new approach for query selection through the exploration of contextual information obtained from social media data, and (2) evaluate whether it is possible to use these queries for monitoring influenza epidemics in South Korea.

## Methods

### Data Sources

#### Epidemiological Surveillance Data

National influenza surveillance data were obtained from the Korean Center for Disease Control and Prevention (KCDC), which routinely collects epidemiological data and national statistics pertaining to influenza incidence, typically with a 1-week reporting lag [12]. We used clinical data and virological data from April 3, 2011 (listed as week 32) to April 5, 2014 (listed as week 14). For clinical data, we used the rates of physician visits for ILI; for virological data, the rates for positive results for the influenza virus in laboratory tests. The data obtained were anonymous and publicly available.

#### Social Media Data

In developing an approach for query selection, we drew on social media data. Social media data were collected from the daily Naver blog (a weblog service offered by the biggest portal site in South Korea [13]) and Twitter posts between September 1, 2010 and August 31, 2013 (3 years), using the social “big data” mining system, SOCIALmetricsAcademy. This system contains social media data crawlers that collect posts from Twitter and the Naver blog. The system also processes text using state-of-the-art natural language processing and text-mining technologies. The Twitter crawler utilizes a streaming application program interface (API) for data collection using the “track keywords” function. We tracked several thousand keywords that were empirically selected and tuned to maximize the coverage of the crawler operating in near real time. We estimated that the daily coverage of the Twitter crawler was more than 80%. The collected posts were fed into a spam-filtering module that checked for posts containing spam keywords written by known spammers. The lists of spam keywords and spammers were semiautomatically monitored and managed. The Naver blog crawler resembles general-purpose Web crawlers, the main difference being that a list of active bloggers for post collection is maintained and automatically expanded. The estimated coverage of the Naver blog crawler was also more than 80%. We applied an extensive spam-filtering process similar to that of the Twitter crawler on the collected blog posts.

The authors and data mining company conducted the search according to the Twitter and blogging website terms and conditions of use. All Twitter and Naver blog posts were publicly available and the information collected did not reveal the identity of the social media users; thus, user confidentiality was preserved.

#### Search Engine Query Data

The query data originated from the search engine on the Korean website, Daum [14]. Although Google is the most-used search engine in the world, it is not dominant in South Korea. Local search engines based on the Korean language, such as Daum, are more widely used than Google. Daum is the second-largest search engine in the portal sites market of South Korea [15]. Because the query data of Korean websites were not publicly available, we sent the list of target queries to Daum and received scaled volume data pertaining to the queries listed. Weekly relative volumes of queries submitted to the search engine between April 3, 2011 and April 5, 2014, were used for analysis. The relative volumes were calculated by dividing the number of each query by the total number of search queries in any given week. The website Daum is written in Korean, thus the submitted queries are primarily in Korean. No information was available that could have potentially revealed the identity of a website visitor; therefore, complete confidentiality was maintained.

### Query Selection

To obtain queries related to influenza that were submitted to the Daum search engine by the Korean population at large, several approaches were applied. Search queries were obtained using the following methods.

#### Seed Keyword for Exploring the Queries

Although “influenza” is the official term used by the KCDC, *dokgam*, *inpeulruenja*, *peulru*, and *sinjongpeulru* are the words typically used in Korea to describe influenza. Since the 2009 pandemic of influenza virus A (H1N1), the term *sinjongpeulru* to describe the new strain of flu has been more popular in Korea than the term influenza A (H1N1). Thus, *dokgam*, *inpeulruenja*, *peulru*, *sinjongpeulru*, “influenza,” and “flu” were defined as seed keywords for exploring the queries. Because Web search queries typically consist of word combinations of an average of two or three terms [16,17], these seed keywords were also used as essential keywords in word combinations.

#### Exploring Influenza-Related Words Through Social Media Data

To obtain search queries related to influenza, we considered the words that usually appear with the word influenza in the accumulated posts submitted to Twitter and blogs. We first conducted synonym processing for the seed keywords of *dokgam, inpeulruenja, peulru*, *sinjongpeulru*, influenza, and flu, and named the resulting app Flu. Then, we investigated the words most likely to be associated with Flu using the accumulated posts during the critical 3-year period (between September 1, 2010 and August 31, 2013). Association analysis was performed to identify tuples of topic keyword and associated keywords. This analysis resulted in a total of 157 associated words.

Certain words associated with influenza were not related to influenza seasons or were not commonly entered into search engines. We excluded keywords that occurred infrequently during the influenza season and those that showed nonsequential patterns in the time series throughout the tracking period. Although relatively rare, we also excluded Korean word combinations written in the form of an incomplete sentence. Therefore, we excluded words considered as inadequate candidates for search query following the keyword filtering; in our first phase, we generated 103 candidate queries of single words or word combinations consisting of seed keywords and/or words associated with influenza as determined using social media data.

#### Identifying Chief Concerns Related to Influenza

Some additional queries related to influenza were obtained through a review of influenza symptoms referring to patients’ chief concerns. The influenza surveillance system of the KCDC defines ILI as the sudden onset of high fever (38°C or greater) accompanied by a cough and/or sore throat. These symptoms, based on the definition of ILI, were included. Additionally, we included influenza symptom definitions used by the Centers for Disease Control and Prevention (CDC) [18] and a consultative committee of medical doctors; this second phase generated 29 candidate queries of single words or word combinations consisting of seed keywords and associated words in reference to chief concerns relating to influenza.

#### Using Web Query Recommendations

Internet search users often require multiple iterations of query refinement to find the desired results from a search engine [16]. Users of search engines can improve their Web search through the help of query recommendations that suggest lists of related queries, allowing users to improve the usability of Web search engines and to access queries that better represent their search intent [17]. We considered queries suggested by keyword recommendations from the Korean websites Daum and Naver In this third phase, entering Flu into the search engines allowed us to identify 75 related queries in the form of single words or word combinations.

### Feature Selection and Prediction Model

We divided the data into training and validation sets. Data from April 3, 2011 to June 29, 2013, were used as the training set for modeling and data from June 30, 2013 to April 5, 2014, were used as the validation set for the model test. Volumes of six seed queries and 146 related queries, obtained after duplicate queries were eliminated from the set of 216 candidate queries, were used for analysis. Before applying the algorithm to each dataset, all data were preprocessed by appropriate transformation and normalization methods. To identify optimal predictors, we applied a least absolute shrinkage and selection operator (Lasso) algorithm. Feature selection can be used to avoid overfitting of irrelevant features and to improve predictive performance (ie, resulting in more rapid and cost-effective predictions) [19,20]. The least absolute shrinkage and selection operator (Lasso) algorithm benefits from a tendency to assign zero weights to irrelevant or redundant features and, hence, is an effective technique for shrinkage and feature selection [21]. Because we aimed to identify predictors of influenza epidemics, feature selection processing was performed at three time points (defined as lag -2, -1, and 0) on the training set portion of the influenza surveillance data using 10-fold cross-validation. We considered all optimal features selected in each lag for model building.

Support vector machine for regression (SVR) was conducted to construct a model predicting influenza epidemics with selected features. Support vector machines, which are represented as one of the kernel-based methods in supervised machine learning, have been applied successfully to classification tasks and, more recently, also to regression [22]. Grid search and 10-fold cross-validation were performed to select the optimal SVR parameter settings, including the penalty parameter *C* and the kernel function parameter such as the gamma for the radial basis function kernel. Ranges of values for grid search can be summarized as follows (elements in each list denote the beginning, end, and number of samples to generate, respectively): penalty parameter *C* (0.01, 10, 0.01); gamma (0.0001, 1, 0.0001). We assessed the root mean square error (RMSE), particular log errors, and the correlation between predicted values and influenza surveillance data using the validation set. All statistical analyses were performed using the R software package (version 3.0.3; R Development Core Team, Auckland, New Zealand).

### Ethics Statement

This study was exempted from ethical review by the Institutional Review Board of Seoul National University.

## Results

A total of 146 queries related to influenza were generated through our initial query selection approach (see Multimedia Appendix 1). Feature selection was performed based on 152 queries including six seed keywords, and optimal features for the prediction of influenza incidence were chosen using 10-fold cross-validation. Table 1 presents the results of feature selection based on ILI surveillance data. Of the 152 queries, 15, 14, and 29 principal features (the total number of features without duplication=36) exhibited the minimum lambda value in lag-2, lag-1, and 0, respectively. The optimal features for the prediction of ILI incidence were derived from queries with reference to social media data (29/36 features), query recommendations (24/36 features), chief concerns relating to influenza (4/36 features), and seed keywords (1/36 features) (Table 1).

**Figure 1:**
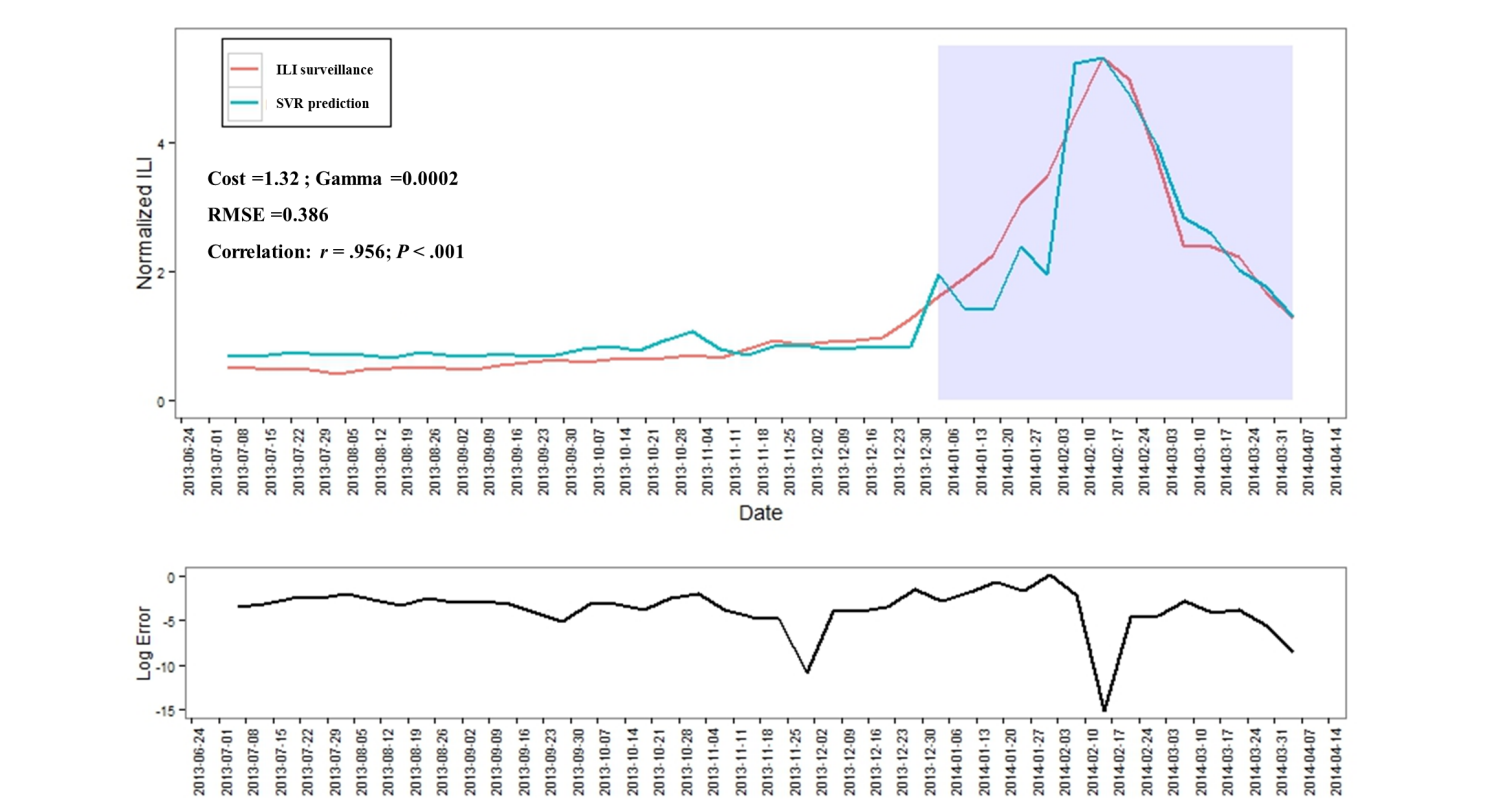
Support vector machine for regression(SVR) prediction and error for influenza-like illness (ILI) surveillance in Korea. This figure shows the performance of the SVR model using the validation set of KCDC surveillance data to predict the next observation. Note: log error=log([obs–exp]2/abs[exp]).

We evaluated the performance of the prediction model, created on the basis of the training set for ILI surveillance, with the validation set. Our results indicated that the SVR model (*C=* 1.32; gamma=0.0002) performed well; the prediction values were highly correlated with recently observed ILI incidence rates (*r*=.956; *P*<.001) (see Figure 1,Multimedia Appendix 2 and Multimedia Appendix 4).

We adopted the same principle with regard to the prediction of virological surveillance as we did with ILI. Table 2 presents the results of feature selection based on virological surveillance data. Of the 152 queries, 28, 26, and 45 principal features (the total number of features without duplication=53) exhibited the minimum lambda value in lag-2, lag-1, and 0, respectively. The optimal features for the prediction of virological incidence were also derived from queries with reference to social media data (42/53), query recommendations (31/53), chief concerns relating to influenza (7/53), and seed keywords (1/53) (Table 2).

**Figure 2:**
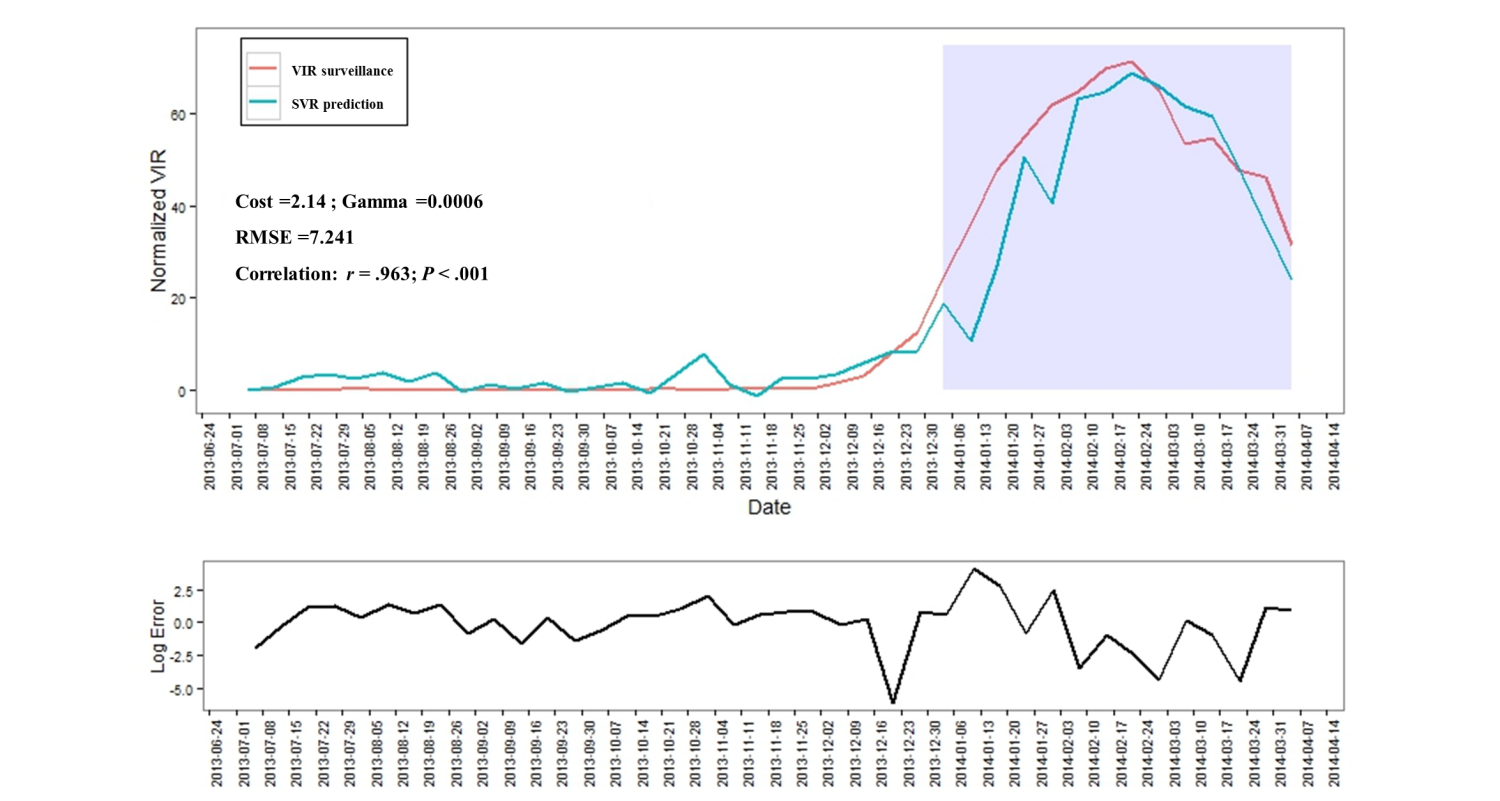
Support vector machine for regression (SVR) prediction and error for virological surveillance in Korea. This figure shows the performance of the SVR model using the validation set of KCDC surveillance data to predict the next observation. Note: log error=log([obs–exp]2/abs[exp]); VIR: virological positive rate.

Figure 2 shows the result of the performance of the prediction model for virological surveillance. The SVR model (*C=* 2.14; gamma=0.0006) performed well; the prediction values were highly correlated with recently observed virological incidence rates (*r*=.963; *P*<.001) (see Figure 2,Multimedia Appendix 3, and Multimedia Appendix 4).

**Table 1:** Optimal features for influenza-like illness surveillance.

Query	Query reference	Coefficient		
		Lag-2	Lag-1	Lag 0
(Intercept)		0.332	0.321	0.497
A hyeong influenza [influenza A type]	Social media; query recommendation	0.745	0	0.109
A hyeong dokgam [influenza A type]	Social media; query recommendation	4.928	20.154	21.503
A hyeong inpeulruenja [influenza A type]	Social media; query recommendation	0.065	0.761	1.127
B hyeong influenza [influenza B type]	Social media query; recommendation	0	0	0.345
B hyeong dokgam [influenza B type]	Social media; query recommendation	0	0.029	1.447
Influenza A	Social media; query recommendation	2.345	0.086	0
Influenza A hyeong [influenza A type]	Social media; query recommendation	1.894	0.927	0.029
Vaccine	Social media	0	0	–0.1151
Geongang [health]	Social media	0.393	0.395	0.109
Dokgamgamyeom [flu infection]	Social media	0.052	0	0
Dokgamgeomsa [flu check]	Social media; query recommendation	4.303	8.893	4.402
Dokgam gyeokrigigan [flu isolation period]	Query recommendation	0	0	0.177
Dokgam gichim [flu cough]	Social media; chief concern	0	0	1.106
Dokgam baireoseu [flu virus]	Social media; query recommendation	0	0	–0.220
Dokgam yeol [flu fever]	Chief concern	0.391	0	0
Dokgam yebang [flu prevention]	Social media; query recommendation	0	0	–0.152
Dokgam yebangjeopjong [flu vaccination]	Social media; query recommendation	0	0	–0.1174
Dokgam ipwon [flu hospitalization]	Social media; query recommendation	0	0	1.470
Dokgam jeonyeom [flu infection]	Social media; query recommendation	0	0	2.569
Dokgam jeonpa [flu dissemination]	Social media; query recommendation	0.547	0.322	0.017
Dokgam pyeryeom [flu pneumonia]	Social media ; chief concern	0	0	0.005
Dokgam hakgyo [flu school]	Social media	0	0.122	0
Dokgam hwanja [flu patient]	Social media	0.066	0	0
Soa dokgamjeungsang [child flu symptoms]	Query recommendation	0.811	0.323	0.135
Sinjongpeulru jeungsang [new flu symptoms]	Social media; query recommendation	55.980	46.156	58.415
Simhangamgi [severe cold]	Social media	0	0	0.031
Eorini dokgamyuhaeng [child flu epidemic]	Query recommendation	0	0	0.002
Onmomi apeum [whole body pain]	Chief concern	0	0.038	0.072
Inpeulruenja geomsa [influenza check]	Social media; query recommendation	0	0.233	0
Inpeulruenja yak [influenza medicine]	Social media; query recommendation	0	0	–0.005
Inpeulruenja yuhaeng [influenza epidemic]	Social media	0	0	0.003
Inpeulruenja jeungsang [influenza symptoms]	Social media; query recommendation	6.254	0	0
Inpeulruenja jeungse [influenza symptoms]	Social media; query recommendation	0	0	0.209
Junggukdokgam [China influenza]	Query recommendation	0	0	–0.056
Tamipeulru [Tamiflu]	Social media: query recommendation	0	0	0.517
Peulru [flu]	Seed keyword	0.621	0.562	0.339

**Table 2:** Optimal features for virological surveillance.

Query	Query reference	Coefficient		
		Lag-2	Lag-1	Lag 0
(Intercept)		–1.459	–3.124	–2.147
A hyeong influenza [influenza A type]	Social media; query recommendation	26.413	18.899	22.579
A hyeong dokgam [ influenza A type]	Social media; query recommendation	0	0	379.041
B hyeong dokgam [ influenza B type]	Social media; query recommendation	6.007	15.324	24.039
B hyeong dokgamjeungsang [ symptoms of influenza B type]	Social media; query recommendation	0	0	0.229
Influenza A	Social media; query recommendation	37.953	25.021	17.449
Influenza ahyeong [influenza A type]	Social media; query recommendation	24.114	19.342	11.426
Gamgibaireoseu [cold virus]	Social media	0	0	4.898
Gamgi pparrinatneunbeop [how to cure flu quickly]	Query recommendation	5.365	4.262	2.343
Gamgiyebang [cold prevention]	Social media; query recommendation	0	0	–0.450
Gamgiyebangbeop [how to prevent a cold]	Social media	–0.155	–2.736	–4.140
Geongang [health]	Social media	4.091	3.562	3.390
Geunyuktong [muscle pain]	Social media; chief concern	0	0	–0.265
Nalssi [weather]	Social media	0	0	–0.111
Dokgam ahyeong [flu A type]	Social media; query recommendation	0	0	22.772
Dokgamgamyeom [flu infection]	Social media	12.236	1.449	0
Dokgamgeomsa [flu check]	Social media; query recommendation	38.254	31.878	0
Dokgam gyeokrigigan [flu isolation period]	Query recommendation	0	0	12.145
Dokgam goyeol [flu high fever]	Social media; chief concern	0	0	1.745
Dokgam gichim [flu cough]	Social media; chief concern	0	0	25.911
Dokgam noin [flu in the elderly]	Social media	0	0	–3.739
Dokgam baireoseu [flu virus]	Social media; query recommendation	0	0	–0.777
Dokgam i [flu child]	Social media	0	0	2.694
Dokgam eorini [flu child]	Social media	0	0	–0.477
Dokgam yebang [flu prevention]	Social media; query recommendation	–2.467	–9.760	–12.191
Dokgam yebanghaneunbangbeop [how to prevent flu]	Query recommendation	0	0	–0.638
Dokgam yuhaeng [flu epidemic]	Social media; query recommendation	0	0	–0.109
Dokgam ipwon [flu hospitalization]	Social media; query recommendation	8.156	0	13.793
Dokgam jeonyeom [flu infection]	Social media; query recommendation	38.184	81.830	9.762
Dokgam jeonpa [flu dissemination]	Social media; query recommendation	2.596	5.613	3.973
Dokgamjusa [flu injection]	Social media; query recommendation	–3.907	0	0
Dokgamjuuibo [flu watch]	Query recommendation	0.883	0.310	0
Dokgam hakgyo [flu school]	Social media	9.268	0	0
Dokgam hapbyeongjeung [flu complication]	Social media	0	0	3.513
Dokgamhwanja [flu patient]	Social media	7.024	5.027	3.205
Dwaejidokgam [swine flu]	Query recommendation	0.358	0	0
Maseukeu [mask]	Social media	8.053	0	0
Momsal [body aches]	Social media; chief concern	0	1.387	3.912
Soa dokgam jeungsang [child flu symptoms]	Query recommendation	4.737	8.058	9.041
Adong dokgam jeungsang [child flu epidemic]	Social media; query recommendation	0	0	–5.273
Eoreun dokgam jeungsang [adult flu symptoms]	Query recommendation	5.156	1.485	0.610
Eolgultongjeung [face pain]	Chief concern	–1.057	0	0
Onmomi apeum [whole body pain]	Chief concern	2.962	3.725	4.791
Uisa [doctor]	Social media	–3.153	–0.436	–0.712
inpeulruenja ahyeong [influenza A type]	Social media; query recommendation	0	8.349	5.837
Inpeulruenja samang [influenza death]	Social media; query recommendation	0	–0.363	–5.193
Inpeulruenja yak [influenza medicine]	Social media; query recommendation	0	0	–0.560
Inpeulruenja jeungse [influenza symptoms]	Social media; query recommendation	3.039	2.051	5.303
Ipwon [hospitalization]	Social media	0	0	–0.213
Joryudokgam [avian flu]	Query recommendation	3.972	4.239	3.492
Tamipeulru [Tamiflu]	Social media; query recommendation	0	65.618	75.462
Pyeryeom [pneumonia]	Social media; query recommendation; chief concern	0	0	–1.288
Peulru [flu]	Seed keyword	15.992	13.406	5.924
Hwanja [patient]	Social media	–4.543	–3.170	–2.922

## Discussion

This study investigated whether search queries have the capacity to enhance the traditional influenza surveillance system in South Korea. To select queries most likely to be associated with influenza epidemics, we adopted an approach that explored contextual information available in social media data. A considerable proportion of optimal features for our final models were derived from queries with reference to the social media data. Our best model for South Korean ILI data included 36 queries and was highly correlated with observed ILI incidence rates. Our model for virological data, which included 53 queries generated through the same principles as the ILI model, performed equally well in terms of its correlation with observed virological incidence rates. Hence, our models for detecting national influenza incidence have the power to monitor changes. These results demonstrate the feasibility of search queries in enhancing influenza surveillance in South Korea.

Created to predict the incidence of influenza throughout the year, including during high- and low-incidence seasons, our model performed as well as previous models that had benefited from full access to search logs to predict influenza incidence using search queries [3,4,6]. Researchers who do not have full access to search logs need to choose the most pertinent queries, but these may be difficult to determine [1]. Our current approach for query selection using social media data appears to be ideal for supporting influenza surveillance based on search query data. First, it may be helpful for obtaining information for query selection because they contain a greater variety of contextual health information, with diverse descriptions of health states. Above all, it may be a more efficient and unobtrusive way to gather health information. Second, an approach using social media data offers clues for understanding such predictors and their weight, which may vary over time. In generating a prediction model using search query data, it is important to note that search queries change over time. An individual’s search behavior changes constantly and keywords submitted by individuals may be influenced by numerous factors, such as media-driven interest or various events [5,23,24]. These changes alter or degrade the performance of search query-based surveillance. The recent Google Flu Trends overestimation can also be understood in the same context [7,8]. Constructing a model that is flexible over time is probably the most difficult, but also the most important, task to complete in the future creation of robust surveillance systems. The systematic exploration of changing predictors in social media data may help to update models based on search queries within a statistical learning framework.

Internet usage is strongly associated with behaviors related to health information seeking and sharing. Some users write expositions about their health through various social media channels, such as blogs and Twitter, while some users leave query logs of health-related questions on the Internet search engines of websites. These types of activities may provide complementary information; it is likely that social media data contain diverse descriptions of personal experiences and information, whereas search engine query data specifically relate to queries, which are submitted for the sole purpose of obtaining information. Starting with studies that have exploited search trends, suggested first in 2006 [25], the notion of detecting influenza activity using Internet-based data has been extended to experimentation with social media data [25]. Thus far, several studies have tried to separately evaluate the scientific potential of each type of novel data for detecting emerging influenza incidence. Although previous empirical studies have reported some significant results, this domain of inquiry is still very much in its infancy [5,23,24] and several limitations pertaining to data sources can be identified [7,8]. Beyond simply conducting experiments to replicate the findings of previous studies using each type of novel data, perhaps it is time to consider a new strategy, one that adopts mutually reinforcing measures of the valuable information contained in each type of data.

We have used query data obtained from Daum, a Korean local website. The market share of Daum is only 17.4% despite being the second-largest search engine in South Korea; nevertheless, our prediction exhibited strong congruence with national ILI incidence rates. Previous research using query data from Daum has found that some cumulative queries selected by means of survey were also strongly correlated with national influenza surveillance data in South Korea between September 6, 2009 and September 1, 2012 [9]. The findings jointly suggest the possibility of developing an influenza surveillance system using a nondominant search engine.

However, changes in Internet usage rates and health information seeking rates may constitute a somewhat central limitation on the use of search query data. Noise from irrelevant information and uncertainty regarding the representativeness of the sample of health information seekers are also significant limitations. These limitations exist in the data used in our study; thus, optimal features of our model may need to be updated over time.

The initial days of an epidemic represent a critical period for health authorities in terms of initiating appropriate interventions. An online surveillance system allows for cost-effective and near real-time monitoring of infectious disease outbreaks through rapid data collection.

Despite several limitations, this study provides further evidence, based on a new approach, for linkages between the use of Internet-based data and the surveillance of emerging influenza incidence in South Korea. We found that Internet-based influenza surveillance that combines search engine query data with social media data has the power to detect influenza outbreaks, exhibiting strong congruence with traditional surveillance data. Such an approach may provide valuable support in preparing for severe pandemics, such as the 2009 influenza A (H1N1) pandemic, and in controlling seasonal influenza epidemics. Furthermore, in an attempt to exploit the complementary nature of two types of data sources, in this study we fused information drawn from social media with a methodology for query-based influenza surveillance. Our results imply that these new data sources can be compatible and complementary in predicting influenza incidence. Our approach indicates that an online surveillance system can play a significant role in detecting infectious diseases such as influenza in near real time before the release of official reports in South Korea.

## Appendix

Multimedia Appendix 1 Queries related to influenza generated by an initial query selection approach.

Multimedia Appendix 2 Support vector machine for regression(SVR) prediction and error for influenza-like illness(ILI) surveillance in Korea.

Multimedia Appendix 3 Support vector machine for regression(SVR) prediction and error for virological surveillance in Korea.

Multimedia Appendix 4 Search for the optimal final model.

## Abbreviations

API: application program interface
ILI: influenza-like illness
KCDC: Korea Center for Disease Control and Prevention
Lasso: least absolute shrinkage and selection operator
RMSE: root mean square error
SVR: support vector machine for regression
