# Supplementary material for: Estimating Influenza Outbreaks Using Both Search Engine Query Data and Social Media Data in South Korea
Source: J Med Internet Res. 2016 Jul 4;18(7):e177. doi: 10.2196/jmir.4955 (PMC4949385; doi:10.2196/jmir.4955)
Supplement: Multimedia Appendix 1 [file jmir_v18i7e177_app1.pdf]

**Table S1.** Queries related to influenza generated by an initial query selection approach

| | | | | | | | |
|---|---|---|---|---|---|---|---|
| | | **Ref. Social media data** | | | | | |
| **Ref. Chief complaint of influenza** | | | | | | | |
| **Query** | **In English** | **Query** | **In English** | **Query** | **In English** | **Query** | **In English** |
| *dokgam yeol* | flu fever | *goyeol* | high fever | *gamgi baireoseu* | cold virus | *dokgam josim* | flu be careful |
| *guto* | vomiting | *geunyuktong* | muscle pain | *gamgi yebangbeop* | how to prevent a cold | *dokgam joeun* | flu good |
| *dokgam guto* | flu vomiting | *gichim* | cough | *geongang* | health | *dokgam jinryo* | flu medical treatment |
| *dokgam mokapeuma* | flu throat pain | *dokgam goyeol* | flu high fever | *nalssi* | weather | *dokgam chiryobeop* | flu treatment |
| *moksseurarim* | sore throat | *dokgam geunyuktong* | flu muscle pain | *dokgam gamgi* | flu cold | *dokgam hakgyo* | flu school |
| *mokibueum* | swollen throat | *dokgam gichim* | flu cough | *dokgam gamyeom* | flu infection | *dokgam hapbyeongjeung* | flu complication |
| *moktongjeunga* | throat pain | *dokgam dutong* | flu headache | *dokgam geongang* | flu health | *dokgam hoheup* | flu breath |
| *momsalgiun* | body aches symptoms | *dokgam balyeol* | flu fever | *dokgam gyeoul* | flu winter | *dokgam hwanja* | flu patient |
| *eolgultongjeung* | face pain | *dokgam komul* | flu runny nose | *dokgam nalssi* | flu weather | *dokgamuisa* | flu doctor |
| *yeol* | fever | *dokgam pyeryeom* | flu pneumonia | *dokgam noin* | flu in the elderly | *maseukeu* | mask |
| *onmomi apeum* | whole body pain | *dutong* | head ache | *dokgam maseukeu* | flu mask | *myeonyeokryeok* | immunity |
| | | *momsal* | body aches | *dokgam myeonyeokryeok* | flu immunity | *baireoseu* | virus |
| | | *balyeol* | fever | *dokgam mok* | flu throat | *Baeksin* | vaccine |
| | | *inhutonga* | sore throat | *dokgam mom* | flu body | *simhan gamgi* | severe cold |
| | | *komul* | runny nose | *dokgam mom sangtae* | flu body state | Vaccine | |
| | | *gigwanjiyeom* | bronchitis | *dokgam byeongwon* | flu hospital | *inpeulruenja yuhaeng* | influenza epidemic |
| | | *pyeryeom** | pneumonia | *dokgam samang* | flu death | *inpeulruenja samangja* | people who died of influenza |
| | | *dokgam momsal* | flu body aches | *dokgam samangja* | people who died of flu | *imsanbu dokgam* | pregnant women flu |
| | | | | *dokgam sangtae* | flu state | *Ipwon* | hospitalization |
| | | | | *dokgam i* | flu child | *Uisa* | doctor |
| | | | | *dokgam yak* | flu medicine | *Hwanja* | patient |
| | | | | *dokgam eorini* | flu child | | |
| | | | | *dokgam imsanbu* | flu pregnant women | | |

**Seed keywords :** *dokgam*(flu), *inpeulruenja* (influenza), *peulru*(flu), *sinjongpeulru*(new flu), influenza, flu

**Table S1.** Queries related to influenza generated by an initial query selection approach (continued)

| Ref. Social media data | | | | | |
|---|---|---|---|---|---|
| Ref. Query recommendation | | | | | |
| Query | In English | Query | In English | Query | In English |
| a *hyeong* influenza | a type influenza | *dokgam jeonyeom* | flu infection | *gamgi pparri natneunbeop* | how to cure a cold quickly |
| a *hyeong dokgam* | a type flu | *dokgam jeonpa* | flu dissemination | *dokgam gamgi chai* | differences between flu and a cold |
| a *hyeong dokgam jeungsang* | a type flu symptoms | *dokgam jusa* | flu injection | *dokgam gyeokrigigan* | flu isolation period |
| a *hyeong inpeulruenja* | a type influenza | *dokgam jeungsang* | flu symptoms | *dokgam pparri natneunbeop* | how to cure flu quickly |
| b *hyeong* influenza | b type influenza | *dokgam chiryo* | flu treatment | *dokgam yuhaengjuuibo* | flu watch |
| b *hyeong dokgam* | b type flu | *dokgam e joeun eumsik* | good food for flu | *dokgam yebanghaneun bangbeop* | how to prevent flu |
| b *hyeong dokgam jeungsang* | b type flu symptoms | *seongin dokgam jeungsang* | adult flu symptoms | *dokgam jambokgi* | flu incubation period |
| b *hyeong inpeulruenja* | b type influenza | *sinjong peulru* | new flu | *dokgam juuibo* | flu watch |
| influenza A | influenza A | *sinjongpeulru jeungsang* | new flu symptoms | *dokgam jeungse* | flu symptoms |
| influenza a *hyeong* | influenza a type | *agi dokgam jeungsang* | baby flu symptoms | *dwaejidokgam* | swine flu |
| influenza B | influenza B | *adong dokgam jeungsang* | child flu symptoms | *soa dokgamjeungsang* | child flu symptoms |
| influenza b *hyeong* | influenza b type | *i dokgam jeungsang* | child flu symptoms | *sinjong inpeulruenja* | new influenza |
| Tamiflu | | *eorini dokgam jeungsang* | child flu symptoms | *sinjongdokgam* | new flu |
| *Gamgi* | cold | *inpeulruenja* a *hyeong* | influenza a type | *sinjongpeulru samang* | new flu death |
| *Gamgiyebang* | cold prevention | *inpeulruenja* b *hyeong* | influenza b type | *sinjongpeulru samangja* | new flu death |
| *Gyeouldokgam* | winter flu | *inpeulruenja geomsa* | influenza check | *sinpeul** | new flu |
| *dokgam ahyeong* | flu a type | *inpeulruenja samang* | influenza death | *eorini dokgamyuhaeng* | child flu epidemic |
| *dokgam bhyeong* | flu b type | *inpeulruenja yak* | influenza medicine | *eoreun dokgamjeungsang* | adult flu symptoms |
| *dokgam geomsa* | flu check | *inpeulruenja yebang* | influenza prevention | *yojeum dokgamjeungsang* | current flu symptoms |
| *dokgam baireoseu* | flu virus | *inpeulruenja jeungsang* | influenza symptoms | *yua dokgamjeungsang* | toddler flu symptoms |
| *dokgam baeksin* | flu vaccine | *inpeulruenja jeungse* | influenza symptoms | *inpeulruenja yuhaengjuuibo* | influenza watch |
| *dokgam sinjongpeulru* | flu new flu | *inpeulruenja chiryo* | influenza treatment | *joryudokgam* | avian influenza |
| *dokgam yebang* | flu prevention | *joryudokgam jeungsang* | avian flu symptoms | *junggukdokgam* | china influenza |
| *dokgam yebangjeopjong* | flu vaccination | *jilbyeonggwanribonbu* | KCDC | | |
| *dokgam yuhaeng* | flu epidemic | *tamipeulru* | Tamiflu | | |
| *dokgam ipwon* | flu hospitalization | *pyeryeom** | pneumonia | | |

**Seed keywords :** *dokgam*(flu), *inpeulruenja* (influenza), *peulru*(flu), *sinjongpeulru*(new flu), influenza, flu
