# Supplementary figures and images for: Estimating Influenza Outbreaks Using Both Search Engine Query Data and Social Media Data in South Korea
Source: J Med Internet Res. 2016 Jul 4;18(7):e177. doi: 10.2196/jmir.4955 (PMC4949385; doi:10.2196/jmir.4955)

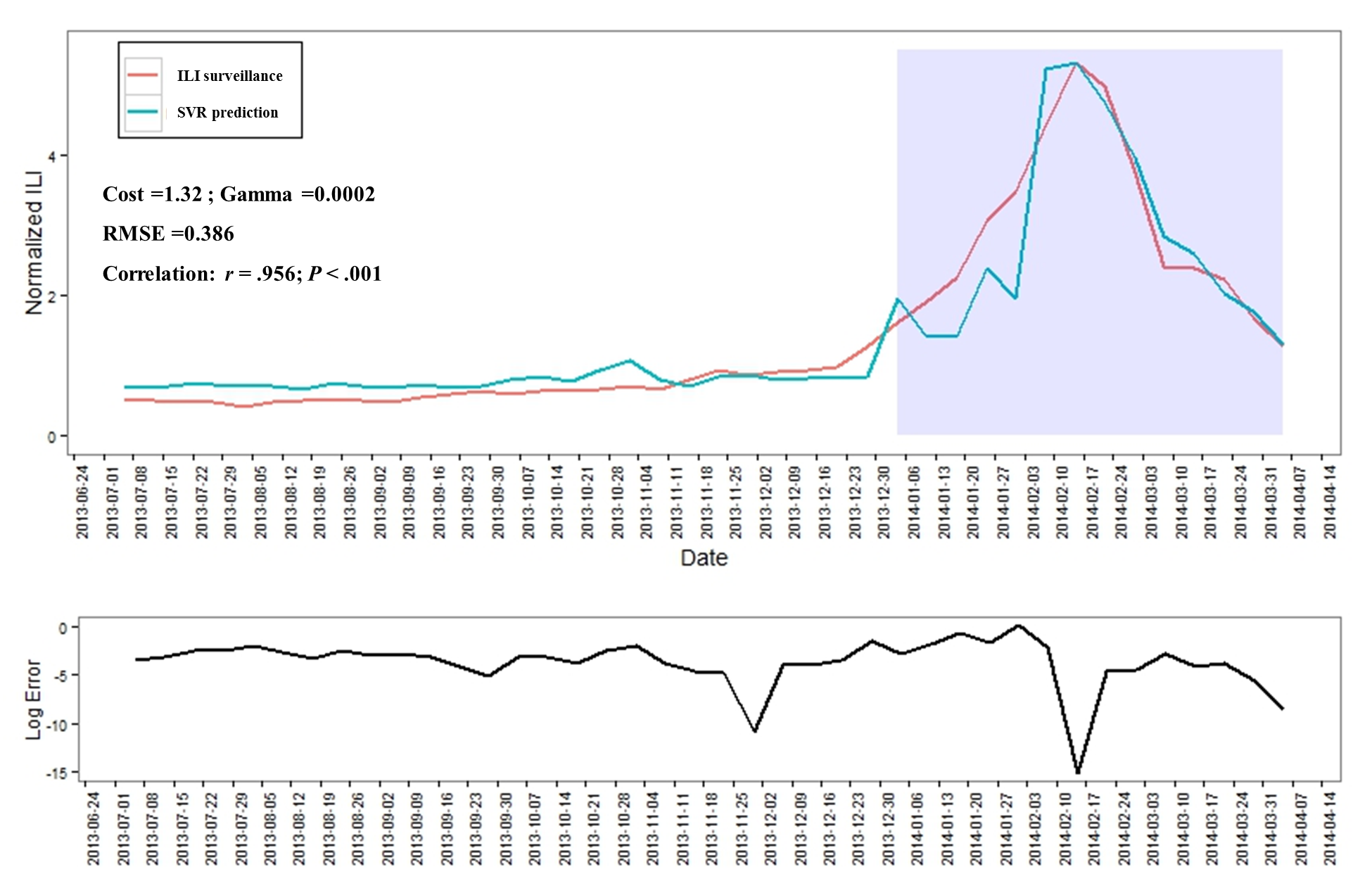

Supplement: Multimedia Appendix 2 [file jmir_v18i7e177_app2.png]

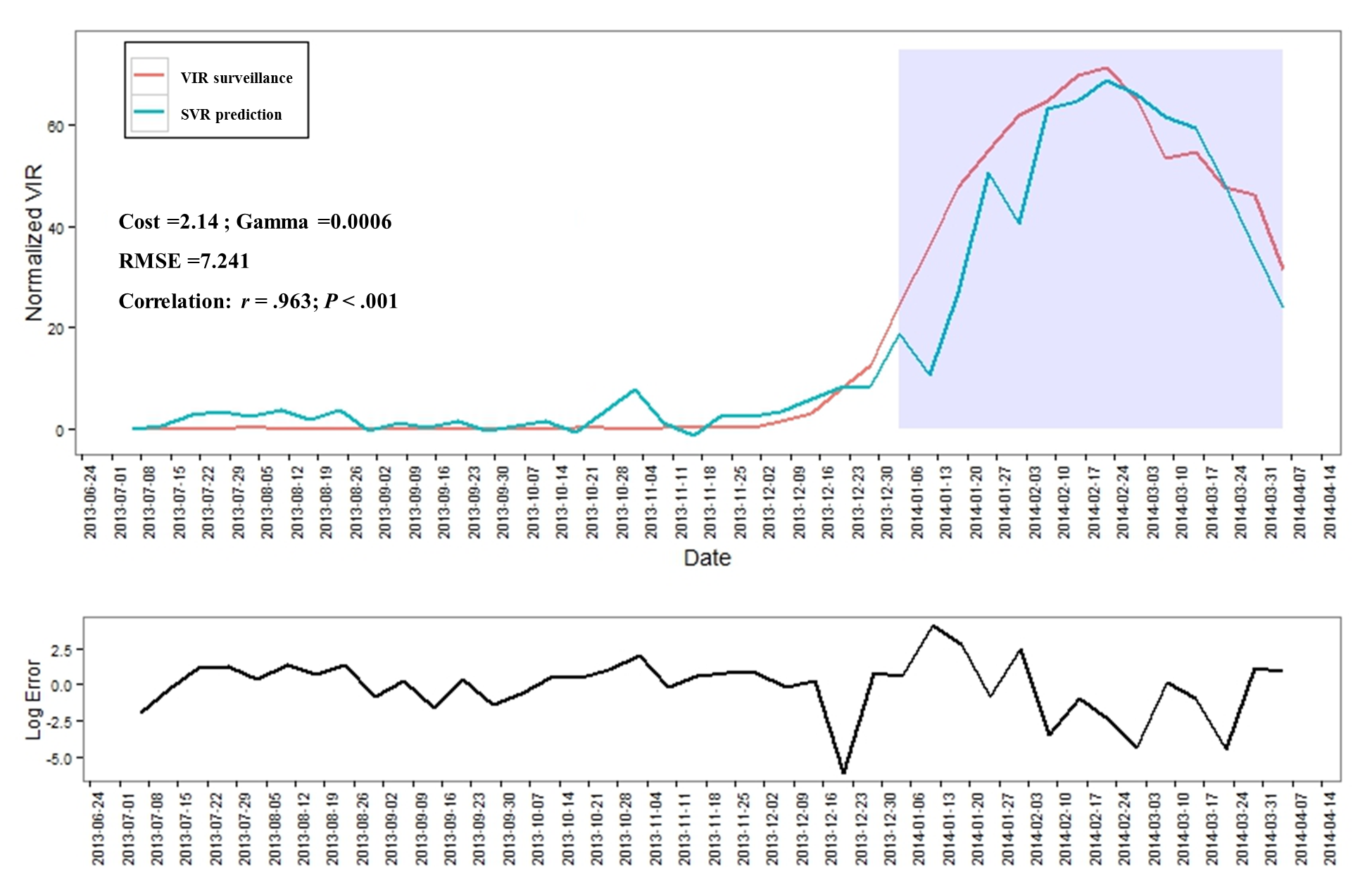

Supplement: Multimedia Appendix 3 [file jmir_v18i7e177_app3.png]
