# Supplementary material for: Estimating Influenza Outbreaks Using Both Search Engine Query Data and Social Media Data in South Korea
Source: J Med Internet Res. 2016 Jul 4;18(7):e177. doi: 10.2196/jmir.4955 (PMC4949385; doi:10.2196/jmir.4955)
Supplement: Multimedia Appendix 4 [file jmir_v18i7e177_app4.pdf]

Table S2 Final SVR Model Comparison according to Data Source

	**Model 1**	**Model 2**	**Model 3[a]**
Main Data Source	Social Media Data (only)	Internet Search Queries (only)	Social Media data & Internet Search Queries
Data Source for Initial Keyword Selection	1. Exploring influenza-related words through social media data	1. Identifying the chief complaint of influenza<br>2. Using the web query recommendation	1. Exploring influenza-related words through social media data<br>2. Identifying the chief complaint of influenza<br>3. Using the web query recommendation
Data Source for and Feature Selection Prediction Model	Twitter/ Naver blog and a combination dataset of both	Queries originate from the search engine of Daum	Queries originate from the search engine of Daum
Model Performance (prediction and error)	1. Correlation between predictions and recent influenza incidence data of 0.92 (p<.001)<br>2. RMSE = 0.554	1. Correlation between predictions and recent influenza incidence data of 0.936 (p<.001)<br>2. RMSE = 0.410	1. Correlation between predictions and recent influenza incidence data of 0.956 (p<.001)<br>2. RMSE = 0.386

[a] Final Optimal Model

Table S3 Final SVR Model Comparison according to Feature selection method

	**Model 3**[a]	**Model 4**
Model Building Process	Initial Query Selection → Feature selection by Lasso → SVR Model building based on best feature index	Initial Query Selection → SVR Model building based on initial query index (without feature selection by Lasso)
Input Feature for Model building	The total number of features without duplication = 36 (Of the 152 queries, 15, 14, and 29 principle features had the minimum lambda value in lag -2, -1, and 0, respectively.)	The total number of features = 155 (full index of initial query)
Model Performance (prediction and error)	1. Correlation between predictions and recent influenza incidence data of 0.956 (p<.001)<br>2. RMSE = 0.386	1. Correlation between predictions and recent influenza incidence data of 0.968 (p<.001)<br>2. RMSE = 0.329
CPU cycle	594.3765/(2.93*10^9) Hz	1773.5184/(2.93*10^9) Hz

[a] Final Optimal Model

Table S4 Model Comparison according to Machine Learning Techniques

	**Model 3[a]**	**Model 5**	**Model 6**
Machine Learning Algorithm	Support Vector Machine for regression (SVR)	Least Absolute Shrinkage and Selection Operator (LASSO)	Random Forest Regression (RFR)
Model Building Process	Feature selection by Lasso → SVR Model building based on best feature index	Feature selection by Lasso → Lasso Model building based on best feature index	Feature selection by Lasso → RFR Model building based on best feature index
Parameter Settings #	Penalty parameter C [0.01, 10, 0.01]; Gamma [0.0001, 1, 0.0001], where elements on list denote beginning, end and number of samples to generate, respectively.<br>(BEST C=1.32, BEST G=0.0002)	Type.measure= ‘mse’<br>BESTLAMDA= lambda.min<br>(BESTLAMDA=0.033127)	MaxTree= 1000<br>BESTNTREE=which.min(RMSE)<br>ntree = BESTNTREE<br>(ntree=596)
Model Performance (prediction and error)	Correlation between predictions and recent influenza incidence data of 0.956 (p<.001);<br>RMSE = 0.386	Correlation between predictions and recent influenza incidence data of 0.545 (p=.0002);<br>RMSE = 10.304	Correlation between predictions and recent influenza incidence data of 0.911 (p<.001); RMSE = 0.776

[a] Final Optimal Model; # To select the optimal SVR parameter settings, 10-fold cross-validation were performed.

Table S5 SVR Model Comparison according to National Influenza surveillance data

	**ILI Surveillance Model (Model 3[a])**	**Virological Surveillance Model**
Main Data sources	Clinical data: rates of physician visits for ILI	Virological data: positive rates for the influenza virus through laboratory tests
Input Feature for Model building	The total number of features without duplication = 36<br>(Of the 152 queries, 15, 14, and 29 principle features had the minimum lambda value in lag -2, -1, and 0, respectively.)	The total number of features without duplication = 53<br>(Of the 152 queries, 28, 26, and 45 principle features had the minimum lambda value in lag -2, -1, and 0, respectively.)
Model Performance (prediction and error)	1. Correlation between predictions and recent influenza incidence data of 0.956 (*p*<.001)<br>2. RMSE = 0.386	1. Correlation between predictions and recent influenza incidence data of 0.963 (*p*<.001)<br>2. RMSE = 7.24

[a] Final Optimal Model
